# Epigenetic regulation of cutaneous T-cell lymphoma is mediated by dysregulated lncRNA MALAT1 through modulation of tumor microenvironment

**DOI:** 10.3389/fonc.2022.977266

**Published:** 2022-08-17

**Authors:** Wei Guo, Guang-Ming Liu, Ji-Yu Guan, Yu-Jia Chen, Yang-Zhi Zhao, Kun Wang, Ou Bai

**Affiliations:** ^1^ Department of Hematology, The First Hospital of Jilin University, Changchun, China; ^2^ Department of Gastroenterology, The First hospital of Jilin University, Changchun, China; ^3^ Key Laboratory of Zoonosis, Ministry of Education, College of Veterinary Medicine, Jilin University, Changchun, China; ^4^ Department of Gastric Colorectal Surgery, The First Hospital of Jilin University, Changchun, China; ^5^ Department of Oncology Hematology, Meihekou Central Hospital, Meihekou, China

**Keywords:** cutaneous T-Cell lymphoma, MALAT1, miR-124, EMT, cancer stem cells

## Abstract

Cutaneous T-Cell Lymphoma (CTCL) is a rare non-Hodgkin lymphoma marked by migration of T-lymphocytes to the skin. It has many subtypes some of which are aggressive with documented metastasis. We investigated a possible role of lncRNA MALAT1 in CTCL cells because of its documented involvement in cancer metastasis. A screening of MALAT1 in CTCL patients revealed its elevated levels in the patients, compared to healthy individuals. For our investigation, we employed HH and H9 CTCL cells and silenced MALAT1 to understand the MALAT1 mediated functions. Such silencing of MALAT1 resulted in reversal of EMT and inhibition of cancer stem cell phenotype, along with reduced cell growth and proliferation. EMT reversal was established through increased E-cadherin and reduced N-cadherin while inhibition of cancer stem cell phenotype was evident through reduced Sox2 and Nanog. CTCL patients had higher circulating levels of IL-6, IL-8, IL-10, TGFβ, PGE2 and MMP7 which are factors released by tumor-associated macrophages in tumor microenvironment. MALAT1 sponged miR-124 as this tumor suppressive miRNA was de-repressed upon MALAT1 silencing. Moreover, downregulation of miR-124 attenuated MALAT1 silencing effects. Our study provides a rationale for further studies focused on an evaluation of MALAT1-miR-124 in CTCL progression.

## Introduction

Cutaneous T-Cell Lymphoma (CTCL) is rather a rare cancer that starts in T-lymphocytes and affects skin. It represents the most common lymphoma of skin (1) with indications of some connections with the industrialization of communities (1). Some of the most common subtypes of CTCL are Mycosis Fungoides (MF), Sézary Syndrome (SS) and the primary cutaneous anaplastic large cell lymphoma (cALCL) which together contribute to almost four-fifths of all diagnosed CTCL cases (2). The incidence of CTCL is a little over 6 cases per a population size of one million persons in the United States (3). There is indication for geographical variation in incidence rate with some Asian populations reporting relatively higher incidence of particularly rare CTCL subtypes (4).

Metastasis-associated lung adenocarcinoma transcript 1 (MALAT1), also known by its alternate name, nuclear enriched abundant transcript 2 (NEAT2), is an oncogenic long non-coding RNA (lncRNA) that has gained lot of interest in recent years (5–7). As suggested by its name, it induces metastasis of cancer cells and is therefore connected with overall increased metastatic potential of different cancers (8) although some research has even suggested its metastasis-suppressing function (9). Thus, according to many researchers, the role of MALAT1 is controversial and may need to be better elucidated through more comprehensive studies (10). Incidentally, the role of MALAT1 in CTCL is almost unknown with little evidence in the literature. This prompted us to undertake this study wherein we planned to study first the relative abundance of this lncRNA in patient samples followed by the study of its mechanism in CTCL cell lines. In its evaluation in CTCL patients, MALAT1 has previously been shown to be elevated (11). To the best of our knowledge this is only report on MALAT1 in CTCL. In view of the controversy surrounding the oncogenic vs. tumor suppressor role of MALAT1 in human cancers, we started with the quantitation of CTCL in serum of CTCL patients in our cohort and we further employed CTCL cell lines HH and H9 to further characterize the role of MALAT1 in CTCL.

## Materials and methods

### Cell culture

HH cells (CRL-2105) and H9 cells (HTB-176) were obtained from ATCC (USA). According to ATCC, HH is a mature T cell line derived in 1986 from peripheral blood of a patient with aggressive cutaneous T cell leukemia/lymphoma while H9 cell line is a clonal derivative of the Hut 78 cell line and the H9 clone was selected for permissiveness for HIV-1 replication. Both the cell lines were cultured in RPMI-1640 medium modified to contain 2 mM L-glutamine, 10 mM HEPES, 1 mM sodium pyruvate, 4500 mg/L glucose, and 1500 mg/L sodium bicarbonate (ATCC, USA). Cells were cultured in incubators with 5% CO_2_.

### Patients and healthy controls

A total of 10 CTCL patients and 8 healthy controls were recruited to the study. Both, CTCL patients and healthy volunteers were enrolled at the the first Hospital of Jilin University. The study involving volunteer and patient recruitment and procedures was approved by the Ethics Committee at the First Hospital of Jilin University (Approval Number 2021-680). Further, the study complied with the standards set by the Declaration of Helsinki, as enforced by the Ethics Committee at the Jilin University. All the patients and volunteers provided a written consent to be part of the study.

### Serum RNA extraction

RNA was extracted from serum of CTCL patients as well as healthy controls, as described in published report (12). First, we extracted the total RNA by mixing 500 μL of serum with 1.5 mL of Trizol reagent (Thermo Fisher Scientific, China). After incubation at room temperature for 5 min, we added 40 μL of chloroform and mixed it well before further incubation for 15 minutes, followed by centrifugation at 4°C for 25 min at 12,000g. At the end of centrifugation, aqueous phase was collected and transferred to a clean and fresh new tube. Then, 1 mL of isopropanol was added and the tubes kept at room temperature for 10 min. Finally, the tubes were further centrifuged at 4°C for 15 min at 21,100 g to pellet the RNA, and the obtained pellet once washed with ethanol.

### MALAT1 expression

We evaluated the expression of MALAT1 by using TaqMan Gene Expression Assay (Thermo Fisher Scientific, China; cat# Hs00273907_s1), following the protocol provided by the company and as earlier described (12). RT-qPCR was performed using the BioRad RT-qPCR machine. GAPDH (cat# Hs02786624_g1) was used as the experimentally verifiable control gene. The calculations were done using the 2^−ΔΔCT^ method.

### Cell proliferation

For cell proliferation/cell growth, we used cell counting Kit-8 (CCK-8) (Sigma, China), which uses WST-8 (2-(2-methoxy-4-nitrophenyl)-3-(4-nitrophenyl)-5-(2,4-disulfophenyl)-2H-tetrazolium, monosodium salt), to produce a water-soluble formazan dye upon bioreduction in the presence of an electron carrier, 1-Methoxy PMS. The CCK-8 solution was directly added on top of the media in the plates with CTCL cells. The number of CTCL cells in individual wells of the 96-well plates ranged from 3000 to 5000, depending upon the cell line, length of incubation and the individual experiment. The principle of this assay is that WST-8 is bioreduced by cellular dehydrogenases to form an orange formazan product which is soluble in normal cell culture medium. The amount of formazan produced is directly proportional to the number of living cells. The WST-8 was added to cells for 4 hours and the color development was stopped by adding 10 μl of 0.1 mol/l hydrochloric acid. O.D. was read to 450 nm using a Shimadzu spectrophotometer.

### qRT-PCR for miRNA and mRNA detection

RNA extractions were carried out using Trizol (Thermo Fisher Scientific, China) and re-suspended in nuclease-treated H_2_O. cDNA synthesis was prepared by the miR-specific, U6 snRNA-specific or oligo-dT primers method using the Superscript II Reverse Transcription kit (Thermo Fisher Scientific, China). Quantitative PCR was performed using BioRad machine. miRNA or mRNA levels were determined, relative to U6 or GAPDH expression using the SYBR Green PCR kit (Thermo Fisher Scientific, China), respectively. Fold change in expression was determined using the formula of 2^-ΔΔCT^.

### MALAT1 and miR-124 silencing

MiR-124 inhibitor, its scramble control and small interference RNAs targeting MALAT1 along with scramble control were obtained from Gene Pharma Co., Ltd. (Shanghai, China). siRNA against MALAT1 as well as miR-124 inhibitors were transfected into CTCL cells using Lipofectamine 2000 (Thermo Fisher Scientific, China) according to the manufacturer’s instructions.

### ELISA

All kits for the ELISA assays were purchased from Enzo Life Sciences (USA). The kits provide all the reagents needed for quantitative assessment of various factors in human serum. The individual kits contain specific antibodies immobilized on microtiter plates. For the assay, serum samples (100 μl) were applied to the individual wells in the provided 96-well plates, and the plates were incubated at room temperature for 1 hour with gentle shaking. At the end of incubation, the contents were emptied out and replaced with 400 μl of wash buffer for washing. The washings were done a total of 3 times and the wash buffer were aspirated out. Then, biotinylated antibody specific to the factor being assayed by specific kits was added to wells and plates further incubated at room temperature for 1 hour with gentle shaking. This was followed by 3x washings and the aspiration of wash buffer every time. Finally, streptavidin conjugated to horseradish peroxidase was added to each wells and the color allowed to develop by shaking the plates gently for 30 minutes at room temperature. 100 μl of stop solution was then added to each well and the O.D. was read at 450 nm using a Shimadzu spectrophotometer.

### Statistical analysis

All results are expressed as mean ± standard deviation. We used Student’s *t*-test or ANONA (One-way) to compare groups. P < 0.05 was considered significantly different.

## Results

### MALAT1 in elevated in CTCL patients

**Figure 1:**
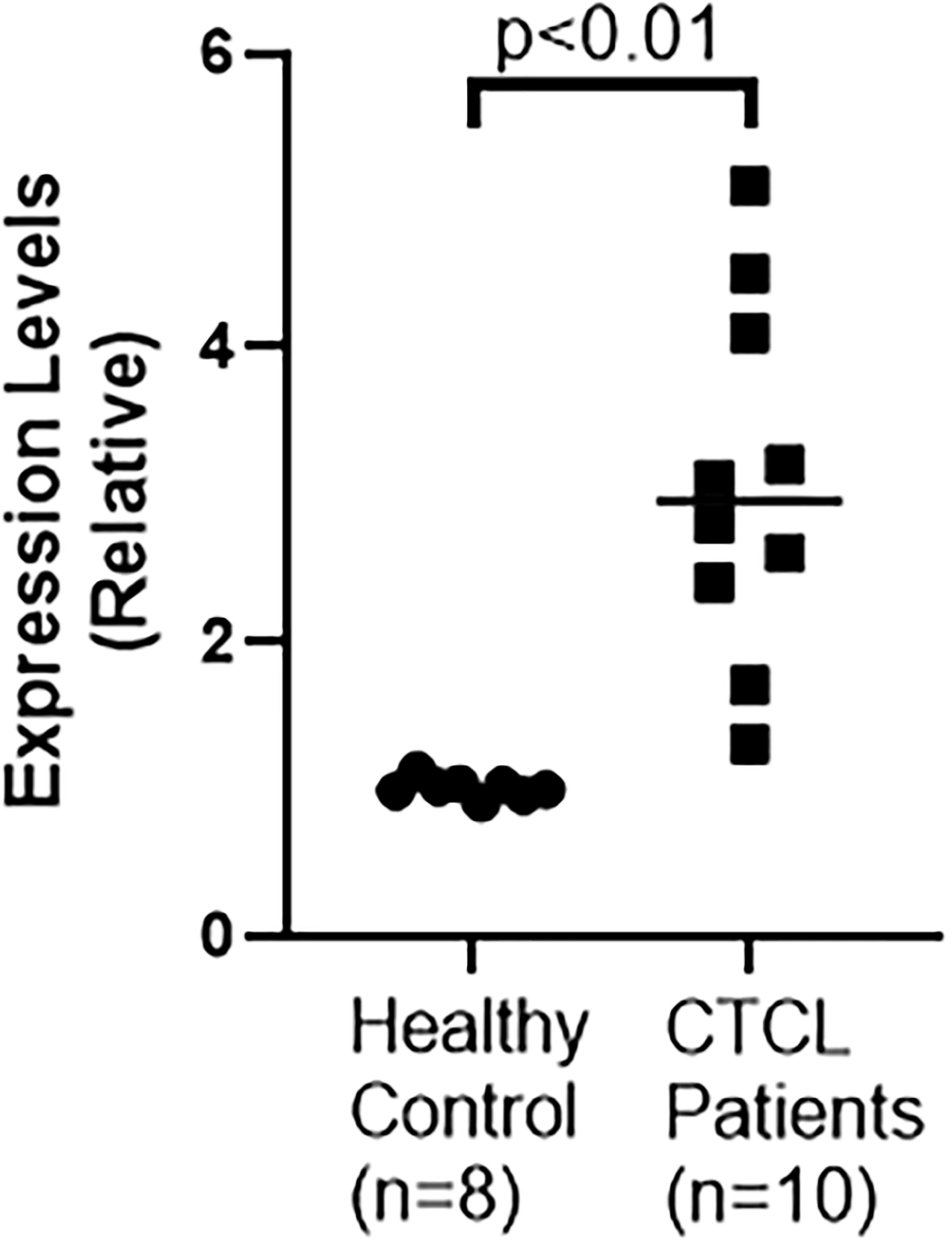
MALAT1 levels in CTCL patients (n=10) vs. healthy controls (n=8).

The status of lncRNA MALAT1 in CTCL patients is not clear and moreover there is quite some controversy regarding the oncogenic vs tumor suppressive role of MALAT1 in different cancers. Therefore, we started with an evaluation of MALAT1 levels in the serum of CTCL patients (n=10), as compared to the levels of MALAT1 in the serum of healthy control volunteers (n=8). In our cohort, we found (**Figure 1**) significantly increased MALAT1 in patients’ serum with a p value of p<0.01. Thus, MALAT1 is clearly overexpressed in CTCL patients, atleast in our cohort.

### MALAT1 silencing affects EMT and cell growth parameters

**Figure 2:**
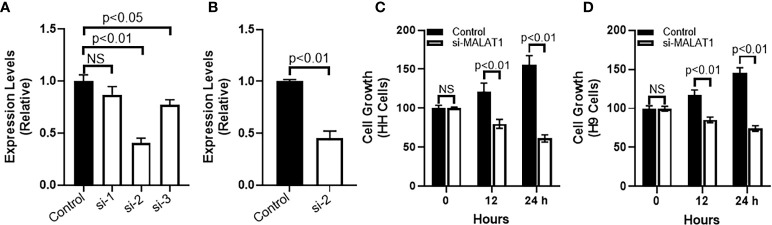
MALAT1 silencing in CTCL cells and its effects on cell growth. **(A)** Different siRNAs were checked for their efficacy to inhibit MALAT1 in HH cells. **(B)** siRNA#2 was further evaluated in H9 cells. Cell growth was assessed by CCK-8 method in HH **(C)** and H9 **(D)** cells. NS is ‘not significant’.

MALAT1 influences cancer metastasis through induction of epithelial-mesenchymal transition (EMT) (13, 14). This made us next evaluate the effect of MALAT1 on EMT in CTCL cells. For this, we started with CTCL cell line, HH, and silenced MALAT1 in this cell line by using specific MALAT1-tergeting siRNA. We first tested three different siRNAs, si-1, si-2 and si-3 (**Figure 2A**) to find the most effective siRNA. Our observations proved that siRNA # 2 was the best in terms of effective silencing of MALAT1 because this particular siRNA reduced the expression of MALAT1 by more than half in HH cells with a p value of p<0.01. In contrast, while one siRNA (siRNA#1) did not significantly reduce MALAT1, another (siRNA#3) was comparatively less effective (p<0.05). Subsequently, we used siRNA#2 for all of the remaining experiments. A screening of siRNA#2 in the other tested CTCL cell line H9 revealed a potent silencing effect of siRNA#2 in these cells as well with significantly reduced MALAT1 levels (p<0.01) (**Figure 2B**).

We further used the siRNA to reduce MALAT1 levels in two different CTCL cells, HH and H9 and first studied the cell growth pattern before studying the levels of EMT markers. Cell growth was measured by CCK-8 method which allows quantification of viable cells and is therefore a reliable test for proliferation and cell growth. Post-silencing of MALAT1, CTCL cells were subjected to CCK-8 assay for 24 hours with reading taken at 1, 12 and 24 hours. We observed that whereas the cells grew normally under control conditions (cells transfected with control siRNA), the ones with silenced MALAT1 were significantly slower in their growth. Same results were seen for both HH and H9 cells (**Figures 2C, D**) with a p- value of p<0.01 for both CTCL cell lines and at 12 and 24 hours post silencing of MALAT1.

**Figure 3:**
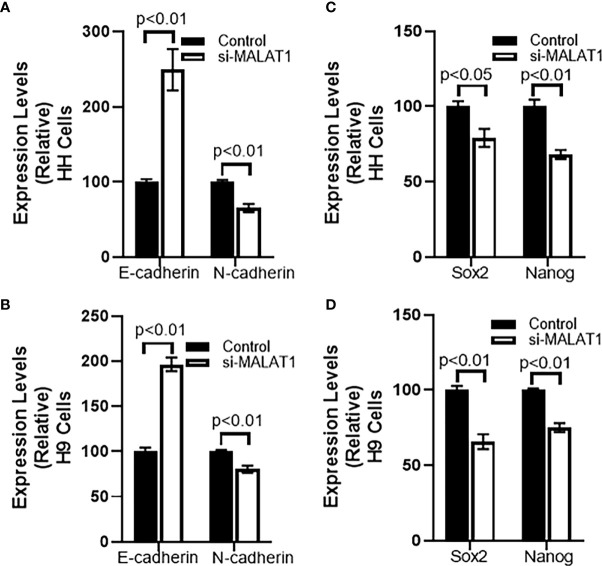
Effect of MALAT1 silencing on EMT and cancer stem cell characteristics of CTCL cells. Epithelial marker E-cadherin and mesenchymal marker N-cadherin was evaluated in HH **(A)** and H9 **(B)** cells upon MALAT1 silencing. Similarly, cancer stem cells markers Sox2 and Nanog were evaluated in HH **(C)** and H9 **(D)** cells upon MALAT1 silencing.

After checking the effect of MALAT1 silencing on cell growth, we next checked for the effect of MALAT1 silencing on EMT. We chose one marker of epithelial phenotype and another marker for mesenchymal phenotype. E-cadherin was our chosen epithelial marker and N-cadherin was our chosen mesenchymal marker. We observed that silencing of MALAT1 increased E-cadherin while reducing N-cadherin in HH cells (**Figure 3A**). These results had a p-value of p<0.01. Similar observations were made in H9 cells as well (**Figure 3B**). We also checked the cancer stem cell markers, Sox2 and Nanog in both of these CTCL cells and observed that both the markers, that we evaluated, were significantly reduced upon silencing of MALAT1 in HH (**Figure 3C**) as well as in H9 (**Figure 3D**) cells.

### Levels of macrophages M2-related factors in CTCL patients

**Figure 4:**
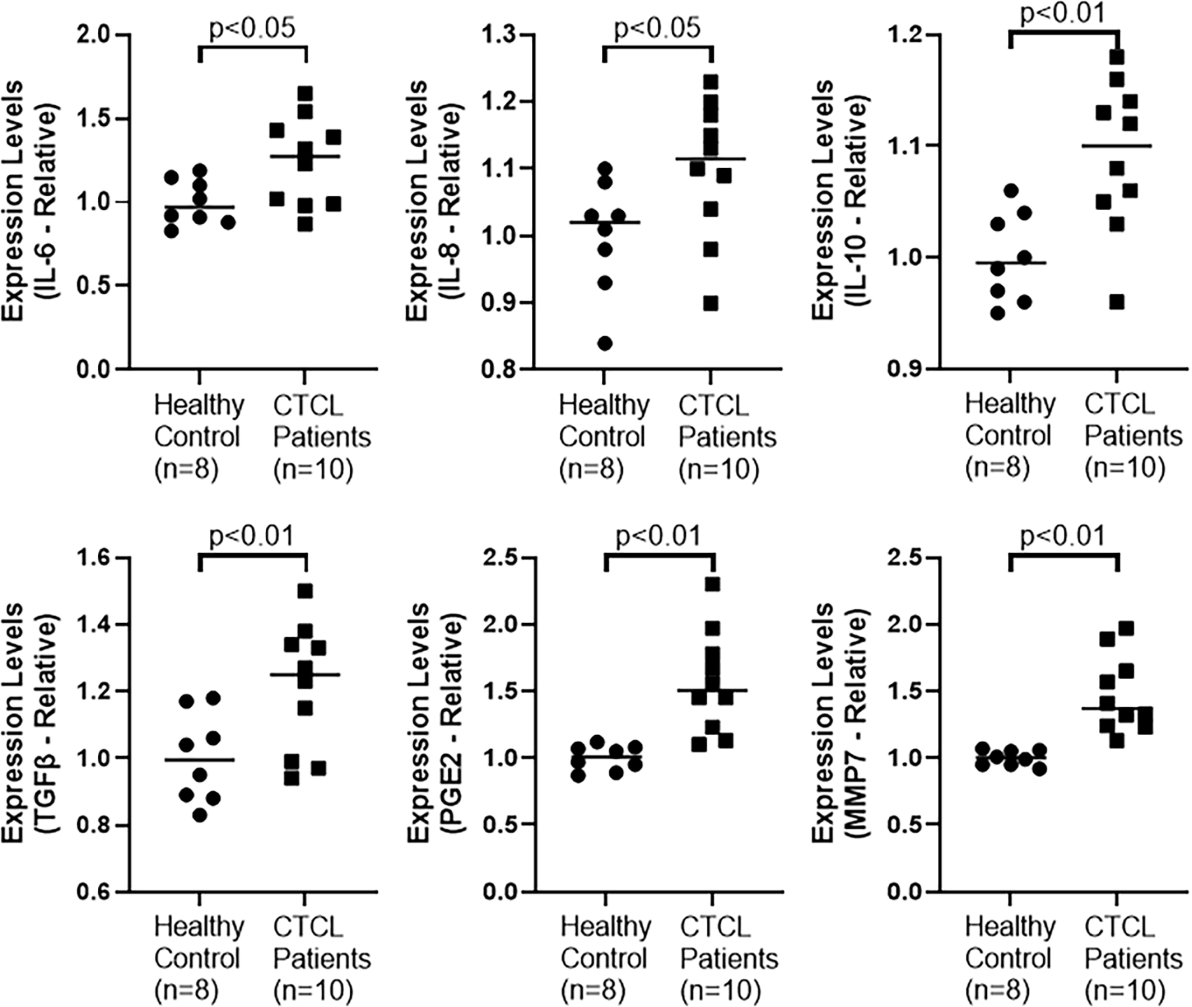
Levels of macrophages M2-related factors in CTCL patients were measured by ELISA, as described in Methods.

Tumor microenvironment plays a very important role in cancer metastasis and tumor-associated macrophages (TAMs), particularly the M2 type play an important role in cancer metastasis. M2 macrophages secrete many factors, such as, IL-6, IL-8, IL-10, TGF-β, PGE2 and MMP7, and these macrophages promote immunosuppression (15) and tumor growth. Therefore, we checked for the levels of these factors in serum of CTCL patients. We observed that all of these factors were significantly elevated in CTCL patients, compared to the healthy control volunteers (**Figure 4**). While IL-6 and IL-8 had p-values of 0.05, IL-10, TGFβ, PGE2 and MMP7 were even more significantly elevated in patients with p-values of p<0.01. Based on these results, it is apparent that the factors related to M2 TAMs are upregulated in the CTCL patients.

### MALAT1 sponges multiple miRNAs in CTCL cells

**Figure 5:**
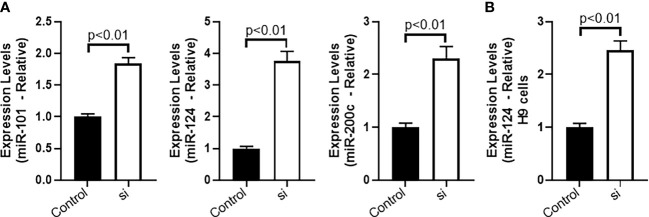
Sponging of miRNAs by MALAT1 in CTCL cells. **(A)** miR-101, miR-124 and miR-200c were quantitated in HH cells followed by evaluation of miR-124 in H9 cells **(B)**.

It is well established in the literature that lncRNAs function *via* sponging of miRNAs (16). miRNAs that have previously been shown to be sponged by MALAT1 and also connected to EMT, namely, miR-101, miR-124 and miR-200c were screened for their possible sponging by MALAT1 in CTCL cells HH. We checked the levels of these three miRNAs in the HH cells with MALAT1 silenced, and observed that all of these miRNAs were upregulated in the cells when MALAT1 was silenced (**Figure 5A**). All the miRNAs had a p-value of p<0.01. However, it was also observed that among the three tested miRNAs, miR-124 was the most upregulated miRNA in HH cells (**Figure 5A**) leading us to further evaluate this miRNA in H9 cells. Similar to our findings in HH cells, we found miR-124 to be significantly elevated in H9 cells (**Figure 5B**). Therefore, we further chose this miRNA for more studies, as discussed below.

### Anti-miR-124 reverses MALAT1 silencing effects

**Figure 6:**
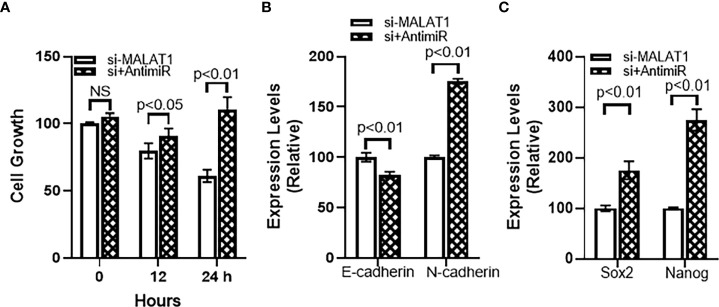
miR-124 reverses MALAT1-silencing effects on **(A)** cell growth, **(B)** EMT and **(C)** cancer stem cells characteristics. si+AntimiR: siMALAT1+Anti-miR-124. NS, not significant.

**Figure 7:**
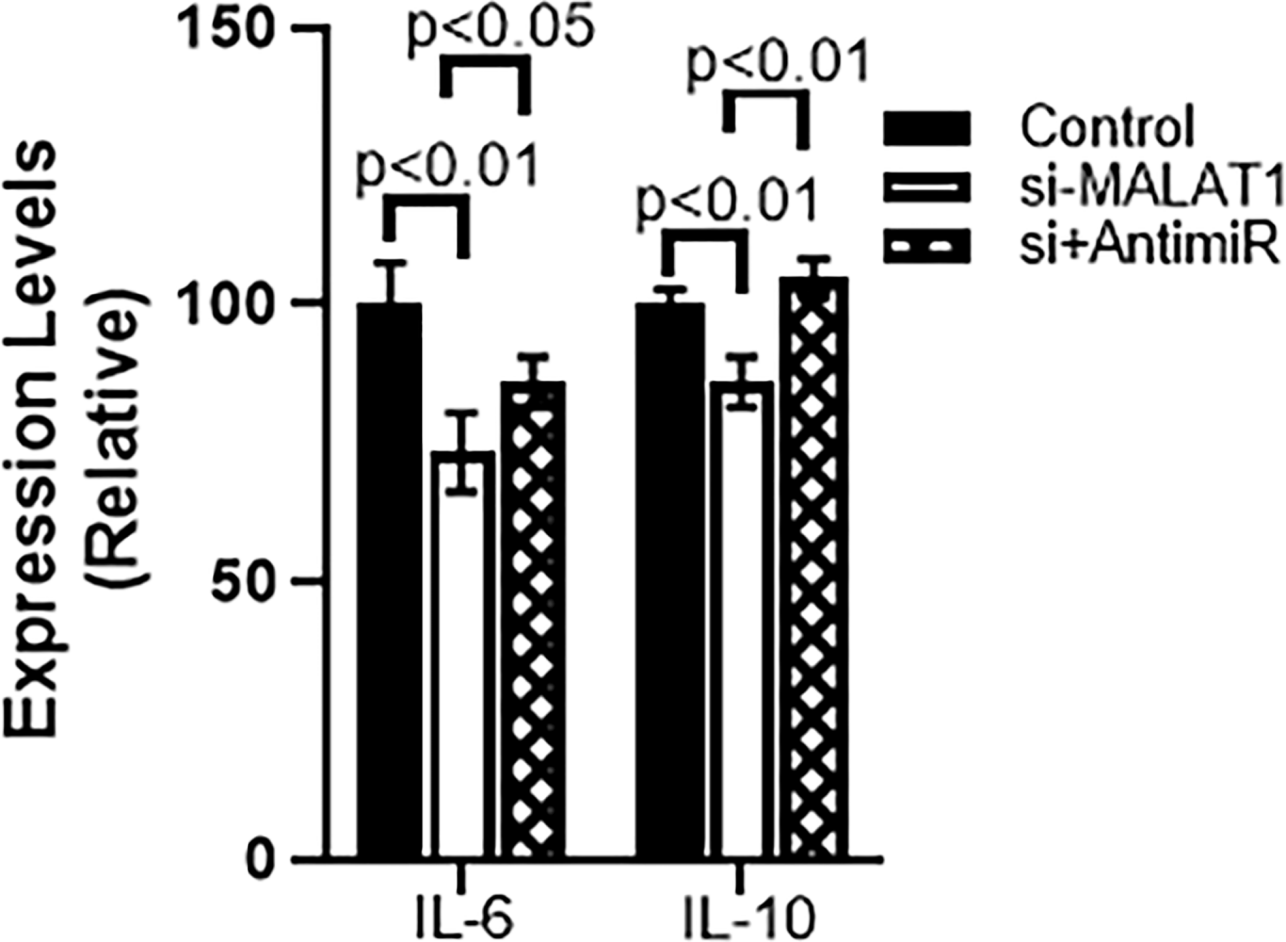
miR-124 reverses MALAT1-silencing effects on IL-6 and IL-10 in HH cells. si+AntimiR: siMALAT1+Anti-miR-124.

Further mechanism was studied because of the observation that miRNA-124 was upregulated in MALAT1 silenced cells. To find a role of miR-124 in MALAT1 functions, we countered the increased miR-124 in MALAT1 silenced cells by silencing miR-124 in these cells. First, we checked the cell growth using CCK-8 method as described above. We observed that when miR-124 was silenced in MALAT1 silenced HH CTCL cells, the reduced proliferation associated with MALAT1 silencing was reversed (**Figure 6A**). Since our results above also found an inhibitory effect of MALAT1 silencing on markers of EMT and cancer stem cells, we also checked for the effect of miR-124 silencing on these markers. We observed that the epithelial marker E-cadherin was downregulated by miR-124 silencing whereas mesenchymal marker N-cadherin was upregulated by miR-124 (**Figure 6B**). When we studied cancer stem cell markers Sox2 and Nanog, we observed that silencing of miR-124 upregulated both the stem cell markers significantly (**Figure 6C**) with a p-value of p<0.01. We also wanted to check the effect of anti-miR-124 on M2 macrophages-released factors. As a proof-of-principle, we checked the levels of IL-6 and IL-10 in control and MALAT1 silenced cells. We observed that MALAT1 silencing significantly reduced both of these factors in HH cells (**Figure 7**). Furthermore, when we silenced miR-124 in MALAT1 silenced cells, the levels of these factors were almost restored (**Figure 7**).

## Discussion

Both lncRNAs and miRNAs belong to the class of non-coding RNAs which were, not very long ago, considered to be ‘junk’ (17). However, now these non-coding RNAs are considered to be very important regulators that determine disease progression, particularly cancer. MALAT1 is one such lncRNA that has now been extensively studied in various different cancers (6, 7, 13, 18) with hundreds of available reports on this lncRNA in the scientific literature. However, there is not much information on this lncRNA in CTCL and that was the primary reason behind our planned study. In the one published report, MALAT1’s role in CTCL was suggested a few years back (11). It was shown that in CTCL MyLA cells, C-C motif chemokine ligand 21 (CCL21) activates mTOR leading to MALAT1 upregulation with observed surge in cell migration (11). Therefore, this early publication on MALAT1 in CTCL supported our current findings by validating an oncogenic role of MALAT1 in CTCL cells. The published report had MyLA cells as the experimental model while our study examined HH and H9 CTCL cells. Combined, it seems like MALAT1 is oncogenic in CTCL cells across multiple cell lines and thus could be an important therapeutic target.

As one of the mechanisms by which MALAT1 can influence CTCL progression, we observed induction of EMT by MALAT1. For establishing this action of MALAT1, we studied two different biomarkers for EMT – E-cadherin and N-cadherin. These markers are representative of two entirely different facets of EMT. While E-cadherin is marker of epithelial phenotype, N-cadherin is a marker for mesenchymal phenotype. During the induction of EMT, cancer cells loose epithelial characteristics and move towards a mesenchymal phenotype. This is marked by loss/downregulation of E-cadherin and gain/upregulation of N-cadherin. In our experiments, we observed gain of E-cadherin and loss of N-cadherin upon MALAT1 silencing. This means that when MALAT1 is downregulated, a reversal of EMT happens. This provides an evidence for a positive correlation between MALAT1 and EMT induction in CTCL cells because upregulation of epithelial marker with simultaneous downregulation of mesenchymal marker is reliable indication of reversal of EMT. The EMT induced by lncRNAs plays an important role in cancer metastasis (19) and accordingly MALAT1-induced EMT has been reported to regulate cancer metastasis (13, 20, 21). Thus, MALAT1 upregulation in CTCL can be related to a more aggressive disease.

In our study, we also checked for the cancer stem cell markers because in our results we observed induction of EMT by MALAT1 and it is known in literature that EMT promotes cancer stem cell phenotype (22). Moreover, MALAT1 has itself been shown to promote cancer stem cell characteristics (23, 24) with silencing of MALAT1 linked to reduced EMT and cancer stem cells phenotype (25), which is in complete agreement with the results that we have presented in our study. In our experiments, we observed reduced levels of stem cells markers, both Sox2 and Nanog in CTCL cells, upon silencing of MALAT1, which is a clear indication that MALAT1 promotes cancer stem cell phenotype as silencing of MALAT1 reduces stem cell markers.

Tumor microenvironment provides a sanctuary for the growth of cancer cells and this is facilitated by mutual interactions between several cellular components of the microenvironment. There is a functional crosstalk between the many different cell types within this microenvironment (26). TAMs represent a major component of tumor microenvironment and they play a very important role in tumor progression through their contribution to evasion of host immune responses (15). TAMs also release many factors such as IL-6, IL-8, IL-10 and TGFβ (27, 28). It was therefore important to find out if these factors are detectable in CTCL patients. Our observations that these factors can be detected in circulation in CTCL patients is a clear indication for the activity of M2-type macrophages in CTCL patients. Further, immunosuppressive activity of TAMs is supported through dysregulated miRNAs (15) and therefore, it was important for us to find miRNA that was central to the MALAT1 activity.

For the identification of miRNAs sponged by MALAT1 in CTCL cells, we focused on three specific miRNAs that have been shown to regulate EMT and also sponged by MALAT1. This was based on the observation that MALAT1 regulates EMT and CSC. All the three tested miRNAs, miR-101, miR-124 and miR-200c regulate EMT (29–31) and there are also reports on their sponging by MALAT1 (32–34). Our observations further add to this knowledge by establishing sponging of these miRNAs by MALAT1 in CTCL cells as well. In our results, we observed miR-124 to be the most dysregulated miRNA when MALAT1 was silenced. This guided us to perform experiments wherein we rescued cancer cell characteristics in MALAT1-silenced cells through targeted dysregulation of miR-124. Since, MALAT1 is oncogenic in our model, we expected the miRNAs sponged by it to be tumor suppressive and that was the case as all sponged miRNAs, including the most affected miRNA, miR-124, are all tumor suppressive miRNAs. miR-124 was upregulated when MALAT1 was silenced and downregulation of miR-124 restored the cancer cell characteristics that would otherwise be seen in MALAT1 expressing cells. Thus, our study established a MALAT1-miR-124 axis in CTCL cells.

Finally, we report in this study that we checked the levels of IL6 and IL10 in CTCL HH cells. For this particular experiment, we had several experimental conditions which included MALAT1 silencing followed by miR-124 downregulation. The rationale for this experiment came from an earlier observation in this study where we saw elevated IL-6 and IL-10 in serum from CTCL patient’s serum. Since manipulation of MALAT1 and miR-124 was not possible in humans, we performed the experiments using CTCL cells as a proof of principle. We were able to show that MALAT1 silencing reduces the levels of these factors and furthermore, downregulation of miR-124 can reverse MALAT1-silencing effects.

Our study thus provides a novel involvement of MALAT1 in CTCL cells. MALAT1 induces EMT and cancer stem cell phenotype and this is facilitated by sponging of miR-124 by MALAT1. Further pre-clinical studies should provide additional verification of this phenomenon with identification of key steps that can targeted for therapeutic benefit.

## Data availability statement

The original contributions presented in the study are included in the article/supplementary material. Further inquiries can be directed to the corresponding author.

## Ethics statement

This study was reviewed and approved by the Ethics Committee of the First Hospital of Jilin University (Approval Number 2021-680). The patients/participants provided their written informed consent to participate in this study.

## Author contributions

WG, G-ML, J-YG, Y-JC performed experiments. WG, J-YG, Y-ZZ and KW analyzed data. WG and G-ML prepared first draft. OB supervised study and edited draft of manuscript. All authors contributed to the article and approved the submitted version.

## Funding

Natural Science Foundation of Jilin Province: 20210101432JC.

## Conflict of interest

The authors declare that the research was conducted in the absence of any commercial or financial relationships that could be construed as a potential conflict of interest.

## Publisher’s note

All claims expressed in this article are solely those of the authors and do not necessarily represent those of their affiliated organizations, or those of the publisher, the editors and the reviewers. Any product that may be evaluated in this article, or claim that may be made by its manufacturer, is not guaranteed or endorsed by the publisher.
